# Medial septal cholinergic mediation of hippocampal theta rhythm induced by vagal nerve stimulation

**DOI:** 10.1371/journal.pone.0206532

**Published:** 2018-11-05

**Authors:** Adam Broncel, Renata Bocian, Paulina Kłos-Wojtczak, Jan Konopacki

**Affiliations:** 1 Neuromedical, Research Department, Łódź, Poland; 2 Department of Neurobiology, Faculty of Biology and Environmental Protection, The University of Łódź, Łódź, Poland; University of Modena and Reggio Emilia, ITALY

## Abstract

**Background:**

Electrical vagal nerve stimulation (VNS) has been used for years to treat patients with drug-resistant epilepsy. This technique also remains under investigation as a specific treatment of patients with Alzheimer’s disease. Recently we discovered that VNS induced hippocampal formation (HPC) type II theta rhythm, which is involved in memory consolidation. In the present study, we have extended our previous observation and addressed the neuronal substrate and pharmacological profile of HPC type II theta rhythm induced by VNS in anesthetized rats.

**Methods:**

Male Wistar rats were implanted with a VNS cuff electrode around the left vagus nerve, a tungsten microelectrode for recording the HPC field activity, and a medial septal (MS) cannula for the injection of a local anesthetic, procaine, and muscarinic agents. A direct, brief effect of VNS on the HPC field potential was evaluated before and after medial-septal drug injection.

**Results:**

Medial septal injection of local anesthetic, procaine, reversibly abolished VNS-induced HPC theta rhythm. With the use of cholinergic muscarinic agonist and antagonists, we demonstrated that medial septal M1 receptors are involved in the mediation of the VNS effect on HPC theta field potential.

**Conclusion:**

The MS cholinergic M1 receptor mechanism integrates not only central inputs from the brainstem synchronizing pathway, which underlies the production of HPC type II theta rhythm, but also the input from the vagal afferents in the brain stem.

## Introduction

Electrical vagal nerve stimulation (VNS) has been used for years to treat patients with drug-resistant epilepsy [1–5]. Although VNS was approved as adjunctive therapy for reducing the frequency of seizures in adults and adolescents, the mechanisms through which VNS modulates activity in the central nervous system are still poorly understood. Interestingly, this technique also remains under investigation as a specific treatment for several other psychiatric and neurological disorders. Among these, VNS is used for the treatment of Alzheimer’s disease [6], depression [4,7,8], schizophrenia [9], migraine [10,11] and central inflammation [12,13]. In addition, VNS has been found to enhance motor and cognitive function in animal models of traumatic brain injury [14], increase alertness [15], enhance the extinction of conditioned fear [16] and alter norepinephrine, dopamine, serotonin and GABA levels in the hippocampal formation (HPC) [17,18]. Interestingly, VNS has also been demonstrated to enhance HPC-induced long-term potentiation (LTP) [19,20] and improve memory in rats and humans [21–23]. The latter findings suggest that VNS may affect memory by enhancing neural plasticity in brain structures associated with memory storage, such as the HPC. This memory processing is related to an increase in the excitation of the hippocampal neuronal network and the presence of a local theta rhythm [24–29]. In agreement with this suggestion, Broncel et al. [30], using several experimental protocols, have recently demonstrated that VNS induced the HPC theta rhythm in anesthetized rats. This was the first direct finding demonstrating the vagal nerve to be involved in central mechanisms underlying oscillations and synchrony in limbic cortex.

The fundamental question arises as to the neuronal substrate and pharmacological profile underlying the effect of VNS on HPC theta. The vagal nerve is a major component of the parasympathetic nervous system and plays a key role in the neuroendocrine-immune axis to maintain homeostasis. It is a mixed cranial nerve consisting of 20% efferent and 80% afferent fibres. The nucleus of the solitary tract (NST), being the main vagal relay site in the brain, receives the most vagal afferents [31]. This nucleus in turn projects to several structures including the locus coeruleus, periaqueductal grey matter, dorsal raphe nucleus, paraventricular thalamic nucleus, amygdala and the medial septum [32–36]. However, there is no direct anatomical projection from the NST to the hippocampal formation [37]. These findings suggest that vagal input might be passed through the NST, and then reaches the HPC probably through the next multi-synaptic pathway that has not been yet described. It seems that the medial septal nucleus and the vertical limb of the nucleus of the diagonal band of Broca (MS/vDBB) is the best candidate for carrying the vagal input from the NST to the hippocampal formation. This region functions as the node in ascending pathways, sending inputs to the HPC [38,39]. It is widely known that MS/vDBB cells act as a pacemaker for discharges of hippocampal neurons responsible for theta production [38,40–42]. In addition, this region is the principal source of strong cholinergic innervation of HPC [38,43–48] which was previously demonstrated to determine the production of HPC type II theta rhythm [38,44,49,50].

The purpose of the present study was to test the hypotheses that: i/ the medial septal region mediates the effect of VNS on HPC type II theta, and ii/ this mediation has a cholinergic profile. Portions of these data have appeared in abstract form [51,52].

## Materials and methods

The studies described below were approved and monitored by Local Ethics Committee for Animal Experiments in Lodz (Permissions Number: 5/LB13/2016). All surgery was performed under anesthesia, and all efforts were made to minimize suffering.

### Subjects and surgical procedure

The data were obtained from 35 male Wistar rats (120–150 g) housed on a 12 h light/dark cycle with free access to water and food. The rats were initially anesthetized with isoflurane (Baxter, Belgium) while a jugular cannula was inserted. Isoflurane was then discontinued, and urethane (0.6 g/ml, Sigma Chemical Co., USA) was administered via the jugular cannula in order to maintain anesthesia throughout the experiment. Anesthesia levels were maintained such that theta field potentials and the transition from theta to large irregular activity (LIA) could occur spontaneously. Body temperature was maintained at 36.5 ± 0.5°C by a heating pad, and heart rate was monitored constantly throughout the experiment.

### Vagal electrode implantation

**Fig 1:**
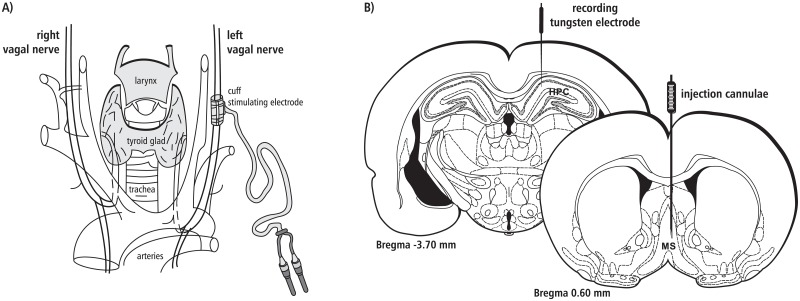
A diagrammatic representation of the electrode implantation arrangement. (A) The platinum-iridium cuff stimulating electrode was positioned on the left vagal nerve. (B) The recording electrode was implanted in the right hippocampal formation and the injection cannula in the right medial septum (see details in the text).

Since it is generally observed that left VNS minimizes potential cardiac effects, such as bradycardia or asystole [4], in the second stage of the procedure, the left cervical-vagal nerve was gently separated from the muscles and then isolated from the carotid artery using glass section sticks. Before implantation of the stimulating electrode, the vagal nerve was moisturized with glycerine. A custom-made platinum-iridium cuff electrode (SPM35 10HY, MicroProbe, USA) was gently placed around the vagal nerve (Fig 1A). The platinum-iridium wire leads were tunnelled subcutaneously for about 2 cm and then connected with the input from a PSIU6 isolation unit (Grass-Astromed, West Warwick, USA).

### Hippocampal electrode implantation

The rats were placed in a stereotaxic frame with the plane between bregma and lambda levelled to horizontal. An uninsulated tungsten wire placed in the cortex, 2 mm anterior to bregma, served as an indifferent electrode, and the stereotaxic frame was connected to the ground. A tungsten microelectrode (0.1–0.9 MΩ) for recording the hippocampal field activity was placed in the right dorsal HPC, in the stratum lacunosum-moleculare (3.7 mm posterior to bregma, 2.0–2.2 mm lateral from the midline and 2.6–2.9 mm ventral to the dural surface [53]; Fig 1B). An AC amplifier (P-511, Grass-Astromed, West Warwick, USA) was used for recording field potentials, with the low filter set at 1 Hz and the high filter set at 0.3 kHz. The mentioned band pass filter was applied to the data prior to any analysis. The field activity was displayed using a digital storage oscilloscope (Tektronix TDS 3014, USA) and a PC computer (Spike 2.7, Cambridge Electronic Design, GB). EEG signals were digitalized by interface (1410 plus, Cambridge Electronic Design, GB) and recorded onto a computer hard disk for subsequent off-line analysis. After the optimal HPC field potential was obtained (theta amplitude of at least 400 μV), the electrode was fixed to the skull with dental cement.

### Medial septal cannula implantation and injections

In preliminary experiments, we did not find evidence for the lateralization of the effects of intraseptal injection of drugs used on hippocampal theta (data not shown). Hence, in the initial experiments, microinjection (26 gauge, 5 ml Hamilton 701N microsyringe) of saline and all agents used were always performed into the right MS (Fig 1B). The coordinates of Hamilton canulae were as follows: 0.6 mm superior from bregma, 0.3 mm lateral from the midline, and 6.5–7.0 mm ventral to the dural surface [53]. All 35 animals receiving the drug injection to the medial septum were divided into six experimental groups (5 or 6 animals each, Table 1): 1/ The animals of group I were administrated with 1 μl of 0.9% NaCl. 2/ The animals of group II were administrated with a local anesthetic, 1 μl procaine hydrochloride (20%). 3/ The animals of group III were injected with cholinergic receptor antagonist atropine sulphate (20 μg/1μl). 4/ The animals of group IV were administrated with cholinergic M1 receptor antagonist dicyclomine hydrochloride (4μg/1 μl). 5/ The animals of group V were administrated with cholinergic M2 receptor antagonist gallamine triethiodide (2 μl/1 μl). 6/ The animals of group VI were injected with selective M1 receptor agonist McN-A343 (0.5 μl/1μl). All drugs used in this study were obtained from the Sigma Chemical Corporation (St. Louis, USA) and injected into the region of the medial septum at the same rate of 1 μl/30 s. The threshold concentration of procaine hydrochloride and atropine sulphate were developed previously [54, 55] and the threshold concentrations of dicyclomine hydrochloride, gallamine triethiodide and McN-A343 were developed in preliminary experiments (data not shown).

**Table 1:** Experimental group and injection compounds.

Group	Place of injection	Compound	Dose/volume	Number of animals
Group I	medial septum	NaCl	0.9%/1μl	5
Group II		procaine hydrochloride	20%/1μl	6
Group III		atropine sulphate	20 μg/1μl	6
Group IV		dicyclomine hydrochloride	4 μg/1μl	6
Group V		gallamine triethiodide	2 μg/1μl	6
Group VI		McN-A343	0.5 μg/1μl	6

### Vagal nerve stimulation

**Fig 2:**
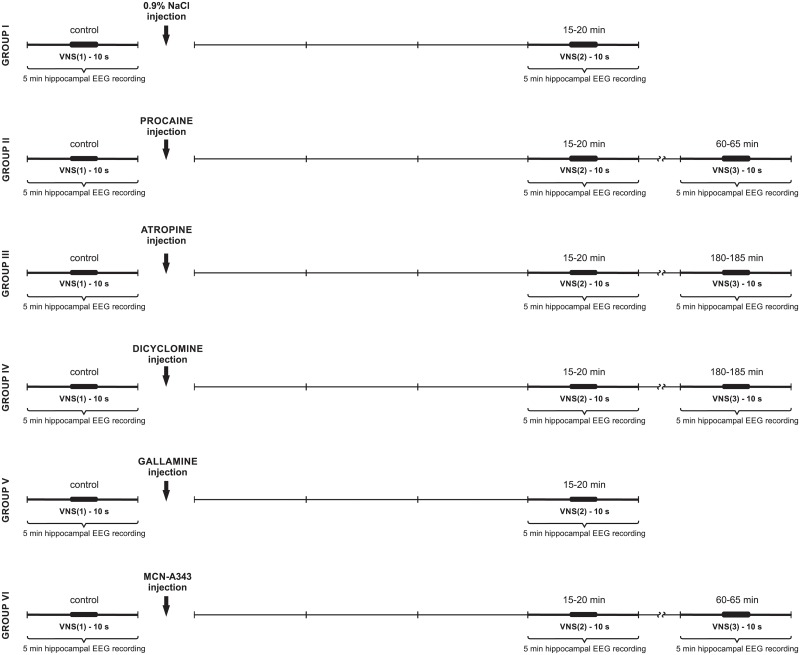
A schematic arrangement of protocols applied in each experimental group (I-VI). Each 5-min pre- and post-VNS period is marked with a thin vertical line. Each 10-s VNS is marked with a thick line (see details in the text).

Based on our previous experiments [30], in the present study, the following square pulse parameters were applied: pulse duration (1 ms), train duration (10 s), frequency (10 Hz) and current intensity of 8 mA. These VNS parameters were previously found to induce a direct, brief effect on HPC field potential, i.e., theta rhythm appearing during vagal stimulation. VNS was delivered through a PSIU6 isolation unit (Grass-Astromed, West Warwick, USA) from an S48 square pulse stimulator (Grass-Astromed, West Warwick, USA). VNS was applied twice or three times, depending on the applied protocol (Fig 2). In each protocol, the medial-septal drug injection was pre-treated by 5 min HPC field recording while 10 s VNS was applied (control). The VNS was always applied at a moment when no spontaneous theta was present in the HPC field potentials.

### Experimental protocols

Initially, the experiments were performed on 39 rats, but only 35 animals were taken into consideration (in four animals, the injection sites in the medial septum were incorrect). Thirty five rats were divided into six experimental groups in which different arrangements of VNS and HPC field potential recording were applied (Fig 2). As shown in Fig 2, in groups I and V, VNS was applied twice: in control, pre-injection time and after the injection, between the 15 and 20 min. In groups II, III, IV and VI, VNS was applied three times: in control, pre-injection time and twice post-injection: between the 15–20 min and then between the 60 and 65 min (group II and VI) or 180 and 185 min (groups III and IV).

### Recording procedure and data analysis

HPC field activity was recorded continuously and analyzed in five-minute panels before drug injection and at strictly defined times after the medial-septal drug injection, (see Fig 2). Power spectra in the 0–50 Hz range for the 10 s HPC field potential recordings obtained during each VNS session were generated using the fast Fourier transform (FFT) algorithm implemented in the Spike 2.7 software package (Cambridge Electronic Design, GB) after a Hanning window was applied to the time series, which were obtained at a sample rate of 100 Hz.

In this study, theta epochs were defined as rhythmic high amplitude sinusoidal waveforms in a strictly defined frequency band (3–6 Hz). These were identified by peaks in the power spectra within that frequency band, and confirmed by visual inspection of the raw EEG traces. Theta frequency for each 10s VNS epoch was then defined as the frequency with maximum power within the 3–6 Hz range, and theta power for each 10s VNS epoch as the peak power value within the same frequency range.

### Statistics

Mean values and standard errors of the mean (± SEM) of two measured theta parameters (power and frequency) obtained during VNS performed before the medial septal drug injection and in successive time periods after the injection were computed and compared. The power and frequency of HPC theta rhythm induced during VNS, before and after the medial-septal drug injections, were subjected to the Shapiro-Wilk test to check the normal distribution of the data. Then, the Mann-Whitney U test was performed (StatSoft Poland).

### Histological procedure

The recording electrode tip location was marked by passing 15 μA current for 14 min (7 min cathodal, 7 min anodal, S48 stimulator; Grass-Astromed, West Warwick, USA). Next, the rat was sacrificed by an overdose of urethane for histological examination. The brain was removed and stored in 10% formalin. Frozen brain sections (30 μm) were taken serially and mounted on glass slides for the reconstruction of the medial septal injection sites and the evaluation of HPC theta recording sites (data not shown).

## Results

**Fig 3:**
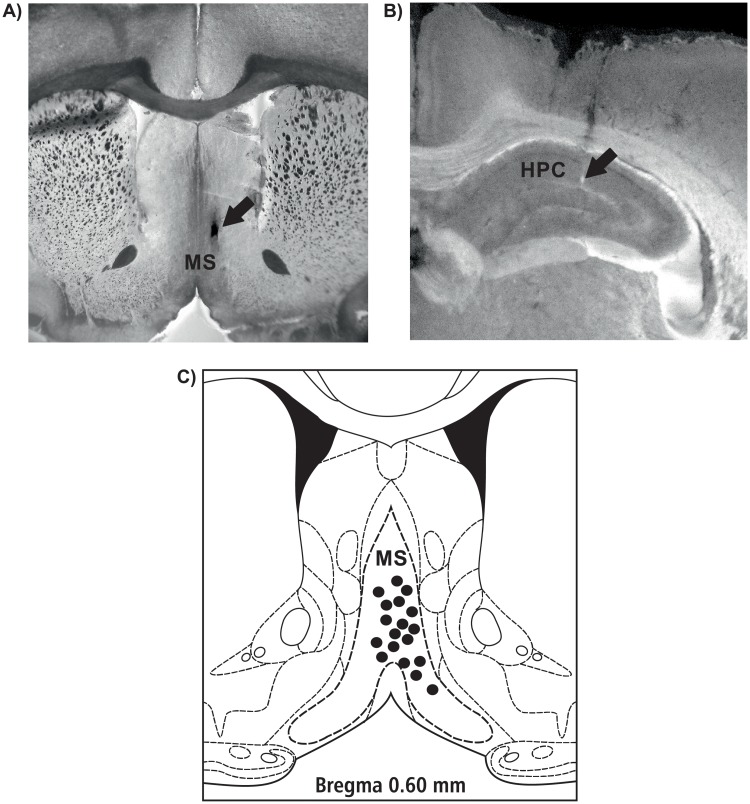
Microphotographs (A and B) and a diagrammatic reconstruction of representative locations of the injection sites in the medial septum in three representative rat preparations of each group (18 sites, C) and representative HPC electrode tip location.

Only data obtained in experiments performed on rats with the correct location of the HPC recording sites and the correct medial septal injection sites were presented in this study (n = 35). Fig 3 provides microphotographs and a diagrammatic reconstruction [53] of representative locations of the injection sites in the medial septum in three representative rat preparations of each group (18 sites) and representative HPC electrode tip location.

### The effect of MS 0.9% NaCl injection on VNS-induced HPC theta rhythm

**Fig 4:**
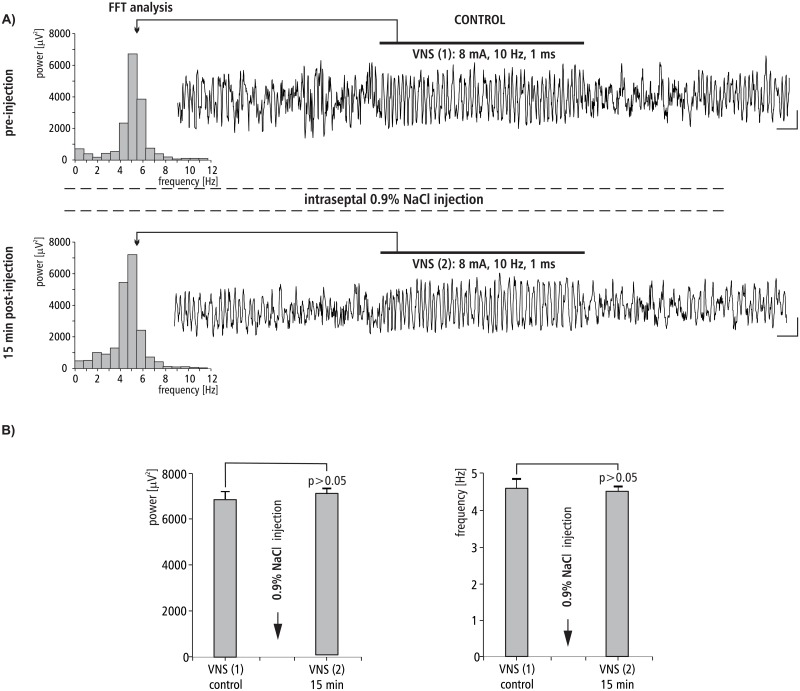
The effect of MS 0.9% NaCl injection on VNS-induced HPC field potential and related power-frequency (FFT) histogram. (A) VNS is marked with a horizontal line. The parameters of VNS are marked below this line. Arrows indicate the power-frequency histograms calculated from the analog examples of hippocampal field potentials taken pre-injection of 0.9% NaCl (control) and 15 min post-injection of 0.9% NaCl during 10 s VNS. Calibration: 1 s, 150 μV. (B) Statistical analysis (Mann-Whitney U test) of mean ± SEM power and frequency (VNS(1) vs VNS(2)).

The effect of intra-septal injection of 0.9% NaCl on VNS-induced type II theta is shown in Fig 4. Fig 4A provides a representative example taken from one animal illustrating the effect of 0.9% NaCl micro-infusion into the MS on VNS-induced HPC type II theta in anesthetized rats. This figure also provides a corresponding power spectrum estimated from each data segment in pre- and post- injection time using the FFT (15 min). Before the MS microinjection of 0.9% NaCl, VNS-induced HPC theta rhythm in the power spectrum had a peak frequency 5.0 ± 0.1 Hz. At 15 min post-injection of 0.9% NaCl, VNS-elicited HPC theta (peak frequency 4.9 ± 0.2 Hz in power spectrum) had a mean power and frequency similar to VNS-induced theta observed in the pre-injection segment (p > 0.05 for power, p > 0.05 for frequency, Mann-Whitney U test; Fig 4B, Table 2).

**Table 2:** Summated statistical details concerning power and frequency of VNS-induced theta rhythm after intraseptal injection of different agents (group I–group VI). Statistical analysis: Shapiro-Wilk and Kruskal-Wallis tests.

Number of VNS	Parameters of theta rhythm	Group					
		Group I	Group II	Group III	Group IV	Group V	Group VI
VNS(1) **control**	power (μV^2^)	6882.0 ± 703.6	6786.0 ± 609.6	6911.8 ± 599.4	9392.6 ± 814.5	7003.9 ± 807.7	7488.0 ± 679.1
		*p > 0*.*05*	*p > 0*.*05*	*p > 0*.*05*	*p > 0*.*05*	*p > 0*.*05*	*p > 0*.*05*
	frequency (Hz)	5.0 ± 0.1	4.6 ± 0.1	4.9 ± 0.2	5.1 ± 0.1	5.2 ± 0.1	4.9 ± 0.2
		*p > 0*.*05*	*p > 0*.*05*	*p > 0*.*05*	*p > 0*.*05*	*p > 0*.*05*	*p > 0*.*05*
VNS(2)	power (μV^2^)	7204.3 ± 633.8	No theta	No theta	No theta	6899.6 ± 747.5	7706.7 ± 728.2
		*p > 0*.*05*				*p > 0*.*05*	*p > 0*.*05*
	frequency (Hz)	4.9 ± 0.2				5.0 ± 0.1	5.0 ± 0.2
		*p > 0*.*05*				*p > 0*.*05*	*p > 0*.*05*
VNS(3)	power (μV^2^)	---------	6211.0 ± 584.6	No theta	No theta	---------	9210.4 ± 812.7
			*p > 0*.*05*				***p < 0*.*01***
	frequency (Hz)	---------	4.4 ± 0.2			---------	5.2 ± 0.1
			*p > 0*.*05*				*p > 0*.*05*

### The effect of MS procaine injection on VNS-induced HPC theta rhythm

**Fig 5:**
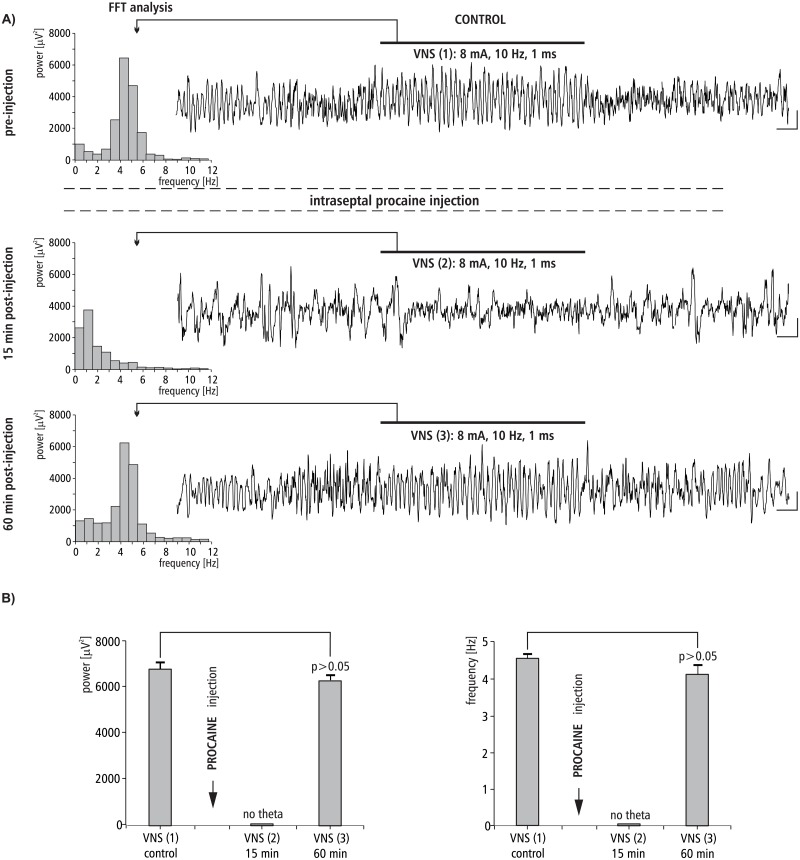
The effect of MS procaine injection on VNS-induced HPC field potential and related power-frequency (FFT) histogram. (A) VNS is marked with a horizontal line. The parameters of VNS are marked below this line. Arrows indicate the power-frequency histograms calculated from the analog examples of hippocampal field potentials taken pre-injection of procaine (control), 15 and 60 min post-injection of procaine during 10 s VNS. Calibration: 1s, 150 μV. (B) Statistical analysis (Mann-Whitney U test) of mean ± SEM power and frequency (VNS (1) vs VNS(3)).

The effect of intra-septal injection of procaine on VNS-induced type II theta is shown in Fig 5. Fig 5A provides a representative example taken from one animal illustrating the effect of procaine microinjection into the MS on VNS-induced HPC type II theta rhythm in anesthetized rats. Fig 5A also provides a corresponding power spectrum estimated from each data segment in pre- and post- injection time using the FFT (at 15 and 60 min). Before the microinjection of procaine into the MS, VNS-induced HPC theta rhythm in the power spectrum had a peak frequency 4.6 ± 0.1 Hz. At 15 min post-injection, VNS no longer elicited HPC theta (absence of a peak in the power spectrum). At 60 min post-injection of procaine, VNS again elicited HPC theta with an power and frequency similar to the control, pre-injection conditions (p > 0.05 for power, p > 0.05 for frequency, Mann-Whitney U test; Fig 5B, Table 2).

### The effect of MS atropine injection on VNS-induced HPC theta rhythm

**Fig 6:**
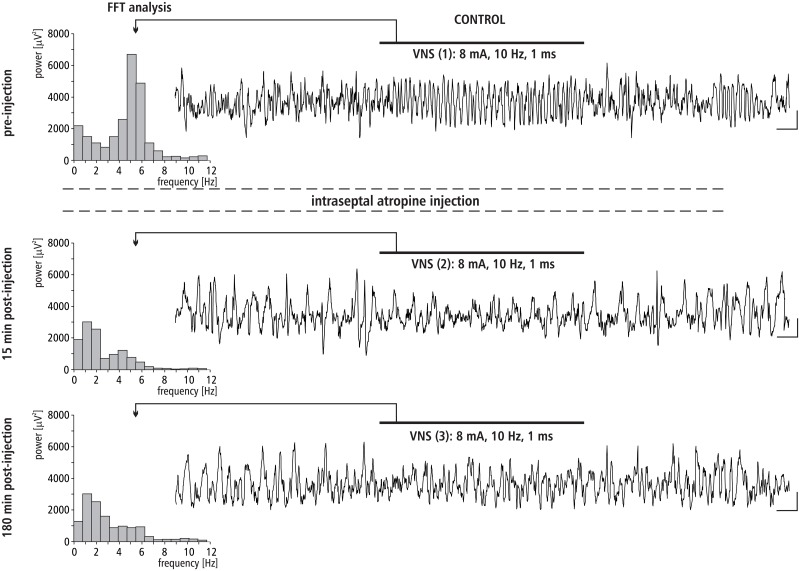
The effect of MS atropine injection on VNS-induced HPC field potential and related power-frequency (FFT) histogram. VNS is marked with a horizontal line. The parameters of VNS are marked below this line. Arrows indicate the power-frequency histograms calculated from the analog examples of hippocampal field potentials taken pre-injection of atropine (control), 15 and 180 min post-injection of atropine during 10 s VNS. Calibration: 1s, 150 μV.

The effect of intra-septal injection of atropine on VNS-induced type II theta is shown in Fig 6. This figure provides a representative example taken from one animal illustrating the effect of atropine micro-infusion into the MS on VNS-induced HPC type II theta in anesthetized rats. Fig 6 also provides a corresponding power spectrum estimated from each data segment in pre- and post- injection time using the FFT (at 15 and 180 min). Before the microinjection of atropine into the MS control, VNS-induced HPC theta rhythm in the power spectrum had a peak frequency 4.9 ± 0.2 Hz. At 15 min post-injection, VNS no longer elicited HPC theta (absence of a peak in the power spectrum). At 180 min post-injection, atropine still completely abolished VNS-elicited HPC theta rhythm (absence of peak in power spectrum of the analog example taken at 180 min post-injection).

#### The effect of MS dicyclomine injection on VNS-induced HPC theta rhythm

**Fig 7:**
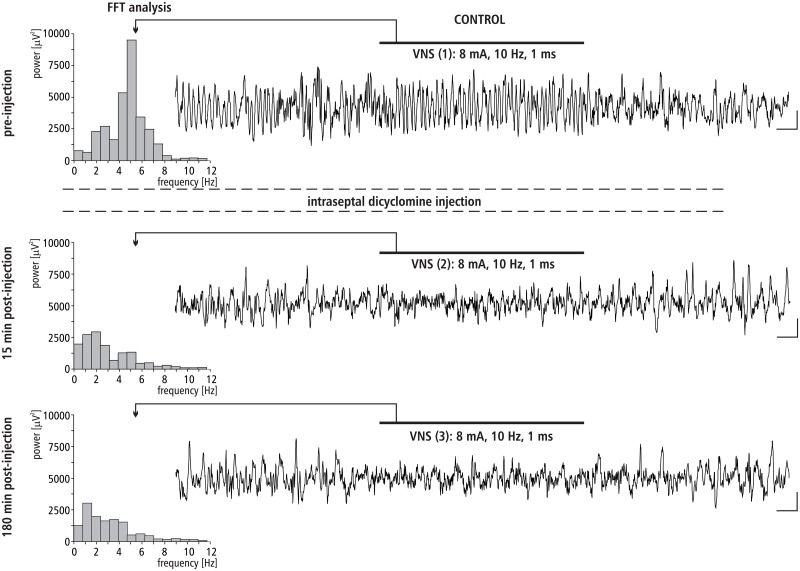
The effect of MS dicyclomine injection on VNS-induced HPC field potential and related power-frequency (FFT) histogram. VNS is marked with a horizontal line. The parameters of VNS are marked below this line. Arrows indicate the power-frequency histograms calculated from the analog examples of hippocampal field potentials taken pre-injection of dicyclomine (control), 15 and 180 min post-injection of dicyclomine during 10 s VNS. Calibration: 1s, 150 μV.

The effect of intra-septal injection of M1 cholinergic antagonist dicyclomine on VNS-induced type II theta is shown in Fig 7. This figure provides a representative example taken from one animal illustrating the effect of dicyclomine micro-infusion into the MS on VNS-induced HPC type II theta in anesthetized rats. Fig 7 also provides a corresponding power spectrum estimated from each data segment in pre- and post- injection time using the FFT (at 15 and 180 min). Before the microinjection of dicyclomine into the MS, VNS-induced HPC theta rhythm in the power spectrum had a peak frequency 5.1 ± 0.1 Hz. At 15 min post-injection, VNS no longer elicited HPC theta (absence of a peak in the power spectrum). At 180 min post-injection, dicyclomine still completely abolished VNS elicited HPC theta rhythm (absence of a peak in the power spectrum of the analog example taken at 180 min post-injection).

### The effect of MS gallamine injection on VNS-induced HPC theta rhythm

**Fig 8:**
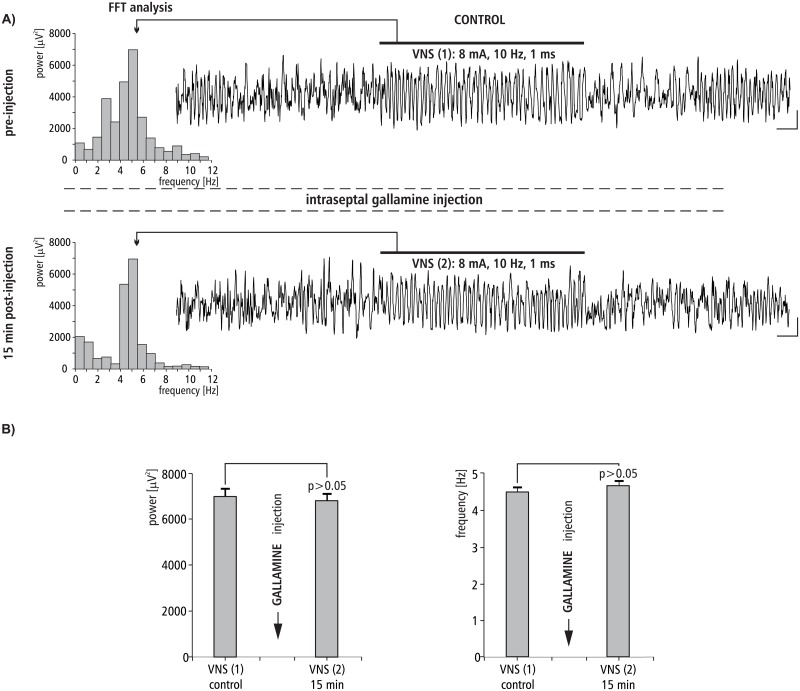
The effect of MS gallamine injection on VNS-induced HPC field potential and related power/frequency (FFT) histogram. (A) VNS was marked with a horizontal line. Parameters of VNS were marked below this line. Arrows indicate the power-frequency histograms calculated from analog examples of hippocampal field potentials taken pre-injection of gallamine (control) and 15 min post-injection of gallamine during 10 s VNS. Calibration: 1s, 150 μV. (B) Statistical analysis (Mann-Whitney U test) of mean ± SEM power and frequency (VNS(1) vs VNS(2)).

The effect of intraseptal injection of M2 cholinergic antagonist gallamine on VNS-induced type II theta is shown in Fig 8. Fig 8A provides a representative example taken from one animal illustrating the effect of gallamine microinjection into the MS on VNS-induced HPC type II theta in anesthetized rats. This figure also provides a corresponding power spectrum estimated from each data segment in pre- and post- injection time using the FFT (at 15 min). Before the microinjection of gallamine into the MS, VNS-induced HPC theta rhythm in the power spectrum had a peak frequency 5.2 ± 0.1 Hz. At 15 min post-injection of gallamine, VNS-elicited HPC theta (peak frequency 5.0 ± 0.1 Hz in the power spectrum) had a mean power and frequency similar to VNS-induced theta observed in the pre-injection segment (p > 0.05 for power, p > 0.05 for frequency, Mann-Whitney U test; Fig 8B, Table 2).

### The effect of MS injection of McN-A343 on VNS-induced HPC theta rhythm

**Fig 9:**
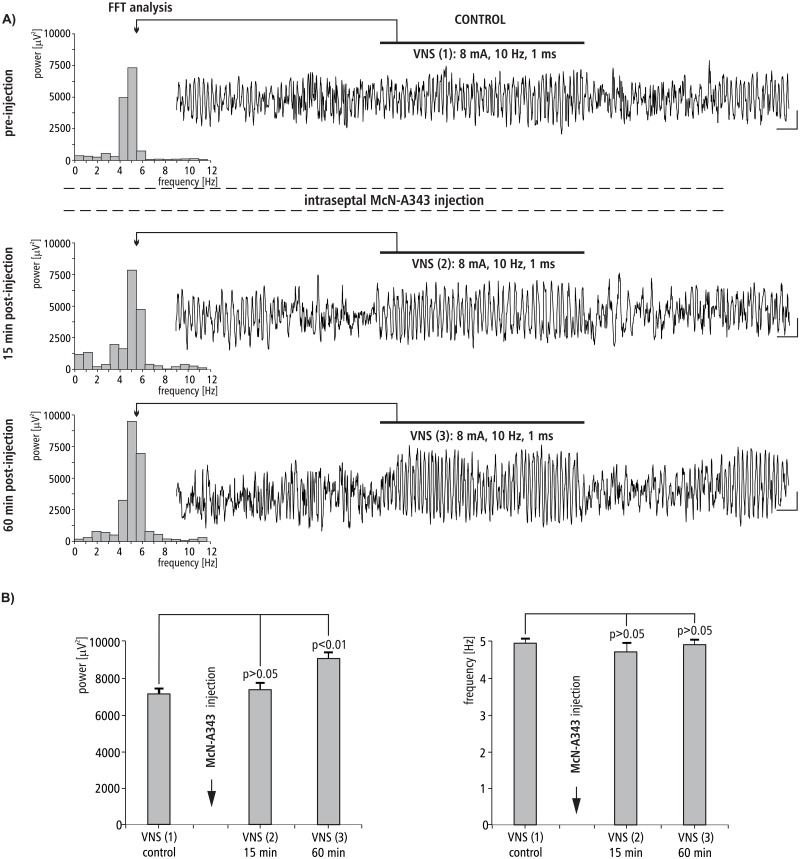
The effect of MS McN-A343 injection on VNS-induced HPC field potential and related power-frequency (FFT) histogram. (A) VNS is marked with a horizontal line. The parameters of VNS are marked below this line. Arrows indicate the power-frequency histograms calculated from analog examples of hippocampal field potentials taken pre-injection of McN-A343 (control), 15 and 60 min post-injection of McN-A343 during 10 s VNS. Calibration: 1 s, 150 μV. (B) Statistical analysis (Mann-Whitney U test) of mean ± SEM power and frequency (VNS(1) vs VNS(2), and VNS(1) vs VNS(3)).

The effect of intraseptal injection of cholinergic M1 agonist McN-A343 on VNS-induced type II theta is shown in Fig 9. Fig 9A provides a representative example taken from one animal illustrating the effect of McN-A343 microinjection into the MS on VNS-induced HPC type II theta in anesthetized rats. This figure also provides a corresponding power spectrum estimated from each data segment in pre- and post- injection time using the FFT (at 15 min). Before the microinjection of McN-A343 into the MS, VNS-induced HPC theta rhythm in the power spectrum had a peak frequency 4.9 ± 0.2 Hz. At 60 min post-injection of McN-A343, VNS-elicited HPC theta (peak frequency 5.2 ± 0.1 Hz in power spectrum) had a similar mean frequency (p > 0.05, Mann-Whitney U test; Fig 9, Table 2). In contrast to the frequency, the power of VNS-induced HPC theta increased, especially 60 min post injection (p ˂ 0.01, Mann-Whitney U test; Fig 9B, Table 2).

## Discussion

The basic experimental model used in the present study to evaluate the central effect of VNS was HPC type II theta rhythm recorded in urethanized rats. We have recently provided the first evidence that, using different protocols and current pulse parameters, VNS produced a very well synchronized HPC type II theta [30].

The theta rhythm is considered to be a “fingerprint” of the limbic cortex [56]. Previous studies demonstrated that theta is involved in a number of physiological regulations, including LTP, learning and navigation, locomotor activity, and sensory-motor integration [57–65]. The studies also showed, both behaviorally and pharmacologically, that theta is not a homogenous field oscillation. In fact, two distinct types of theta rhythms have previously been distinguished: anesthetic-resistant and cholinergic-mediated type II theta at a frequency range of 3–6 Hz, and anesthetic-sensitive, probably serotonin-mediated type I theta (7–12 Hz), which is related to motor behaviors [38, 66–72]. This type of theta depends on the non-cholinergic pathway that arises from the neocortex and cingulate cortex and reaches the HPC via the entorhinal cortex [69].

Recently, Larsen et al. [73,74] using a model of freely moving rats (i.e. type I theta), demonstrated that VNS slowed theta rhythm and decreased its power. In contrast to Larsen et al. [73,74], in the present study, we tested type II theta rhythm which typically is observed in anesthetized rats. The generation of the HPC type II theta is determined by structures of pontine region, hypothalamus and basal forebrain which form the ascending brainstem hippocampal synchronizing pathway [39,43,48,75,76]. The medial septum is considered to be a nodal point of this pathway [44]. On the basis of the above-mentioned data, it seems that in addition to the different model used and different VNS parameters and protocols applied previously [73,74], the difference in neuronal substrate between type II and type I theta could additionally determine a completely different response of HPC rhythmic field potential to VNS in the previous experiments [73,74]. Larsen et al. [73,74] had decisive arguments to suggest that VNS induces a decrease in hippocampal excitation since, in addition to the decrease in amplitude and power of type I theta rhythm, these authors observed decreased efficacy in synaptic transmission. Considering our data concerning type II theta, which appears when the animal is still or anesthetized, we would not go so far as to suggest that VNS induces inhibition of the hippocampal neuronal network. Quite the opposite. The presented data actually indicates the excitation of the hippocampal formation during VNS, as previously suggested [30]. This suggestion is also supported by earlier findings that VNS potentiates hippocampal LTP and enhances hippocampal synaptic transmission in freely moving and anesthetized rats [19,20,23].

The medial septum, is widely accepted to be the pivotal extrinsic regulator of theta rhythm occurring in the limbic system [43,48,77]. The main function of the MS/vDBB is the distribution of inputs to the cingulate cortex, entorhinal cortex, and hippocampal formation, i.e. limbic structures in which well synchronized, local theta field potentials are observed. Rhythmic outputs from the medial septum area act as a “pacemaker” for those structures, inducing theta rhythm [38,39,48,50,67,76,78–82].

In the light of the above-presented data, new and important findings regarding the neuronal substrate underlying HPC type II theta rhythm emerged from the present study. The results of the second experiment, that temporal inactivation of the MS by the local anesthetic procaine reversibly abolishes VNS-induced HPC theta, clearly demonstrate that medial septum integrates not only central inputs from the brainstem synchronizing pathway which underlies the production of HPC type II theta rhythm, but also input from the vagal nerve.

The pharmacological profile of the MS involved in the VNS effect on HPC rhythmic field potentials was evaluated in the remaining experiments described. The experiments conducted in the group III and IV, with use of the MS injection of atropine, the nonselective muscarinic receptor antagonist and dicyclamine, the selective M1 antagonist, suggest that the M1 receptor subtype is involved in the medial-septal mediation of the VNS effect on hippocampal field potential since both agents irreversibly abolished VNS-elicited HPC theta. This suggestion was proved in the next experiments conducted with use of gallamine, the selective antagonist of M2 receptors. Specifically, the MS injection of this agent did not affect the HPC theta rhythm induced by VNS. The definitive confirmation of M1 receptor profile of VNS-induced type II theta was provided in the experiment with the use of McN-A343. This selective M1 receptor agonist was found to enhance the VNS-induced HPC theta power. The described cholinergic profile of VNS-induced type II theta rhythm supports earlier pharmacological findings concerning this type of theta [38,50,67,83–85].

Although the involvement of the muscarinic M1 receptor subtype in the central pharmacological mechanism underlying VNS-induced HPC type II theta was proved in the present experiments, the possible participation of other noncholinergic receptors cannot be discounted. Previous electrophysiological and neurochemical data suggested that the modulatory effect of VNS on hippocampal LTP may involve the central noradrenergic systems. It was earlier pointed out that VNS potentiates noradrenaline (NE) release in the hippocampus [8,17,18] and NE was also demonstrated to increase the discharge rate of HPC pyramidal and granular cells [86,87]. Interestingly, it was also shown that blockade of the dorsal ascending noradrenergic bundle abolished septal elicitation of HPC theta rhythm [88]. On the other hand, NE has never been demonstrated to affect hippocampal theta-related cells, which underlies this field potential [67]. Additionally, the bath perfusion of the hippocampal formation slices with NE did not elicit theta oscillation and did not even alter the production of cholinergic-induced theta rhythm in the HPC slice preparations [89].

Finally, one more issue should be addressed. The other possible explanation for the observed effect of VNS on HPC type II theta rhythm is that VNS may cause changes in peripherally released stress-related hormones, which, in turn, may affect hippocampal function through a mechanism not related to the medial septal area. For example, corticosterone was earlier demonstrated to induce the release of hippocampal acetylcholine [90] leading to the increase of the theta amplitude [91]. However, it should also be pointed out that the release of the corticosterone takes a relatively slow time and VNS-induced theta rhythm appeared in the present study immediately during stimulation.

In summary, the present data provide the first evidence for the role of the medial septum in the mechanism of the effect of VNS on hippocampal formation type II theta rhythm in rats. In addition, the present experiments, with the use of cholinergic muscarinic agonist and antagonists, demonstrated for the first time the involvement of muscarinic M1 receptor subtype in the medial septal mediation of VNS-induced HPC theta.
